# Mitigating risks from hydraulic fracturing-induced seismicity in unconventional reservoirs: case study

**DOI:** 10.1038/s41598-022-16693-3

**Published:** 2022-07-22

**Authors:** Gang Hui, Zhangxin Chen, Ping Wang, Fei Gu, Xiangwen Kong, Wenqi Zhang

**Affiliations:** 1 State Key Laboratory of Petroleum Resources and Prospecting, China University of Petroleum, Beijing, China; 2 Department of Chemical and Petroleum Engineering, University of Calgary, Calgary, AB Canada; 3 Research Institute of Petroleum Exploration and Development CNPC, Beijing, China

**Keywords:** Seismology, Petrol

## Abstract

The recent remarkable increase in induced seismicity in Western Canada has been largely attributed to hydraulic fracturing in unconventional reservoirs. The nucleation of large magnitude events has been demonstrated to be closely linked to site-specific geological and operational factors. A mitigation strategy of fracturing-induced seismicity concerning both factors has not been well investigated. In this paper, a comprehensive investigation of risk mitigations from induced seismicity is conducted based on the formation overpressure, distance to Precambrian basement, proximity to faults, fracturing job size and safe hydraulic fracture-fault distance. It is found that the middle-south region near Crooked Lake is an optimal region for fracturing operations with low formation pressure, a great distance to the basement and relatively fewer pre-existing faults. A field case study suggests that fracturing operations of three new horizontal wells are successful with low magnitude induced events and with high production performance, demonstrating the applicability of a comprehensive approach of seismicity risk mitigations. Such an approach can be applied to other field cases to mitigate the potential fracturing-induced seismicity in unconventional reservoirs.

## Introduction

**Figure 1:**
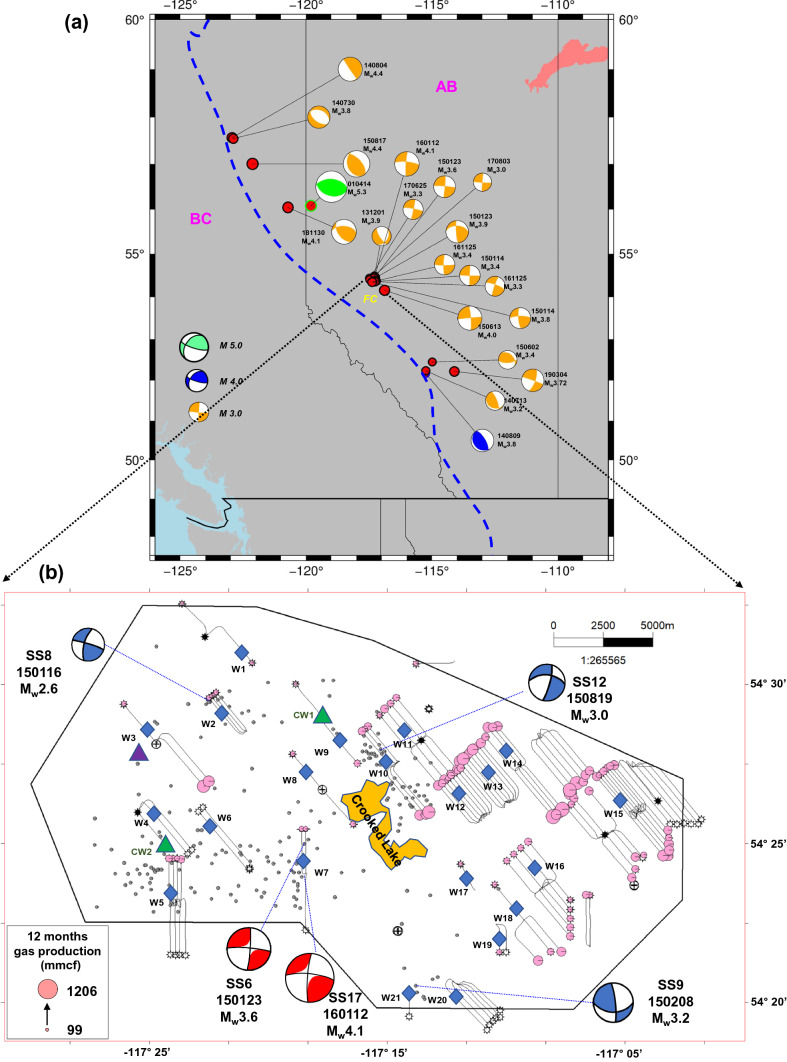
(**a**) Map of recorded induced seismicity in the Western Canada Sedimentary Basin (WCSB). The blue dashed line shows the mountain deformation margin. Red circles show some recorded earthquakes, and the beach balls denote their focal mechanisms of HF-induced (orange), tectonic-related (green), and EOR-induced (blue) events^11,23^. (**b**) Map of induced seismicity and fracturing horizontal wells near the Crooked Lake region. The gray circles show the recorded induced earthquakes. The magnitude-scaled beach balls denote the focal mechanisms of five mainshocks^13,23^. The pink circles show the 12-month shale gas production of horizontal wells. Crooked Lake is marked with a yellow polygon. Two green triangles denote the coring wells drilled for the petrophysical experiments. The blue diamonds represent fracturing horizontal wells with available treatment data. The purple triangle marks the straight well drilled into the Cambrian formation. The black line is the boundary of the available 3D seismic survey.

In recent decades, the remarkable increase in induced seismicity in the Western Canada Sedimentary Basin (WCSB) has been largely attributed to the hydraulic fracturing (HF) operations in unconventional reservoirs in this basin (Fig. 1a)^1–6^. A commonly referenced definition of seismic risk is an estimation of the mean probability (over space and time) of the occurrence of a seismic event with a certain magnitude within a given time interval. Based on the traffic light system implemented by the Alberta Energy Regulator (AER), operators in Alberta must invoke a mitigation strategy if 4.0 > M_L_ (i.e., local magnitude) > 2.0 events are induced during HF operations, whereas suspending operations immediately if M_L_ > 4.0 events are nucleated^7^. Despite this policy constraint for fracturing operations for risk mitigation, many large-magnitude events have been reported during and after HF operations in Western Canada. Statistically, 6% of HF operations targeting the Duvernay formation are related to induced seismicity with moment magnitude *M*_w_ > 3 in the WCSB^8^. The nucleation of such large-magnitude HF-induced seismicity has been demonstrated to be closely linked to site-specific geological, geomechanical, and operational factors, including formation overpressure, the vertical distance to the basement, the lateral distance to carbonate reef margins, the content of shale and total organic carbon, the critical stress state of faults, and the size of the fracturing job^9–14^. Therefore, mitigating risks due to induced seismicity is urgent when performing HF operations in the WCSB.

Based on the factors that control HF-induced seismicity, many mitigation strategies have been proposed to reduce potential seismicity risks. For example, a geology-based optimization of a fracturing site is crucial in mitigating future seismicity risks. Several parameters are relevant for optimizing a site, including ensuring that there is a relatively low formation pore pressure, a large distance to the basement and reef margins, and low shale and total organic carbon content^10,11,14^. In view of operational factors, increasing the distance between the hydraulic fracture and a fault, which can mitigate a potential hydraulic fracture–fault communication during and after stimulation, may also mitigate the risk of potential seismic activities^3,15,16^. However, this strategy rests largely on identifying pre-existing faults before HF operations, which usually requires high-resolution 3D seismic reflection data covering the area of interest^16^. Another operational control strategy is to reduce the fracturing job size (e.g., by decreasing the injection rate or injection volume). However, reducing the fracturing job size can result in a decrease in the stimulated reservoir volume, which can adversely influence the performance of shale gas or oil production in the WCSB^17,18^. Therefore, in this case, a balance should be made between production performance and seismicity mitigation via a comprehensive analysis of the field situation. In addition, adjusting other factors, such as the wellbore orientation of the horizontal fracturing wells and the viscosity of the fracturing fluids, should also be considered as potential mitigation strategies^3,19^. Moreover, the traffic light system implemented by the AER has been utilized to monitor fracturing treatments, which has also shown applicability in risk mitigation^20^.

In this paper, a comprehensive investigation of risk mitigation for HF-induced seismicity is conducted based on field studies near Crooked Lake. Data from well completion, well logging, and core experiments of associated wells and regional 3D seismic data were collected as integrated datasets. A geological model was then established by incorporating the integrated data into a block model. This model depicts the properties of the formation, rocks, faults, and fractures. Based on the spatiotemporal features of the induced seismicity and real-time treatment data from fracturing horizontal wells, an in-depth investigation of geological susceptibility (formation overpressure, distance to the basement, and proximity to pre-existing faults) and operational susceptibility (fracturing job size and safe hydraulic fracture–fault distance) was performed. Finally, new fracturing wells were drilled and fractured with optimal fluid injection within the safe region to keep the seismicity risks low.

## Field background and datasets

The study area is near the Fox Creek (FC) region in Alberta, Canada (Fig. 1a). The west region of the study area has been quiescent historically and then has been moderately active recently since 2013 (Fig. 1b). To explore the shale gas reservoirs in this area, 127 horizontal wells were stimulated by multistage hydraulic fracturing to target the Duvernay Formation (Fig. 1b). This formation was deposited in the Late Devonian, and liquid-rich organic shale gas was widely distributed^21,22^. Based on a statistical correlation of data from well logs and an experimental analysis of core samples from coring wells, the Duvernay formation in the region studied was found to be buried at a depth of 3272–3631 m below the surface (true vertical depth). The average formation thickness is about 39 m, with a range of 37.4–43.3 m. Petrophysical results from two coring wells show that the average effective porosity and average permeability are 3.84% and 131 nD (nanodarcies), respectively. Rock–Eval tests suggest that the mean total organic carbon content is 3.1%. X-ray measurements indicate that the mean shale content is 31.8%. The details of the experimental results of core samples from two coring wells are collected for reservoir property evaluation (see Supplementary Table S1).

Treatment data from 127 horizontal wells were obtained from a well-completion database. The first 12 months of shale gas production data are employed in this work to determine areas with high potential as shale gas reservoirs (Fig. 1b). Based on the statistics of the treatment data within the region studied, the cumulative 12-month gas production per well ranged from 99 to 1206 million cubic feet (MMCF) with a mean of 536 MCF, where 1 million cubic feet is 28,317 m^3^. The average injection volume of the fracturing fluid and the average mass of placed proppants per well were 45,657 m^3^ and 6303 t, respectively. In contrast, the mean number of fracturing stages and mean horizontal length were 33 and 2285 m, respectively.

Data for historical seismicity of *M*_w_ ≥ 2.5 up to 31 January 2020 were obtained for the region studied from the Composite Alberta Seismicity Catalogue (www.inducedseismicity.ca/catalogues, last accessed on 1 September 2021). Figure 1b is a map of these events, where five large-magnitude-induced events are shown. Their focal mechanisms were derived from prior works^13,23^. Note that the west region is more susceptible to induced seismicity and has less production potential, whereas the east region is virtually seismicity-quiescent, with higher production performance. The distribution features of induced events and shale gas production will guide the site’s optimization for drilling new horizontal wells in the study region.

## Methodology

The workflow for assessing the susceptibility to HF-induced seismicity is as follows. First, data from well completion, well logging, and core experiments of associated wells and regional 3D seismic data were collected as integrated datasets from publicly available resources. A geological model of the region studied was then established by incorporating the combined data into a block model that depicts the properties of the formation, rocks, faults, and fractures. Then, based on the spatiotemporal features of the induced seismicity and real-time treatment data from fracturing horizontal wells, an in-depth investigation of the geological and operational susceptibility was conducted for the region studied.

Specifically, the formation overpressure, vertical distance to the Precambrian basement, and spatial distance to pre-existing faults were selected as the important parameters for characterizing the geological susceptibility to HF-induced seismicity^9–11,24^. Additionally, the safe distance between a pre-existing fault and the fracturing site and the optimal fracturing job size (e.g., fluid injection volume, horizontal length, and the number of fracturing stages) were determined based on the relations between known induced seismicity events and fracturing treatment data for the region studied. Finally, based on a comprehensive analysis of the geological and operational susceptibility, a mitigation strategy is proposed for selecting fracturing sites and for optimizing fracturing job sizes in the region studied.

### Geological susceptibility to induced seismicity

#### Formation pore pressure

The formation overpressure has been demonstrated to be an important parameter in HF-induced seismicity^9^. For critically stressed faults, a small additional pressure perturbation during or after fracturing stimulations can cause such faults to slip and may trigger large-magnitude-induced seismicity events^25^. We show five cases in Fig. 1b as an example. Three approaches are usually utilized to estimate the formation pore pressure in a region. The first employs the steady pressure at the end of stage completion during fracturing stimulation of horizontal wells to estimate the formation pore pressure^26^. The second utilizes field monitoring tests of reservoir pressure^9^. The last one uses the Eaton method to predict the formation pore pressure via an integration of the stress, hydrostatic pressure, and sonic log data^27^, which is expressed by

1 $${P}_{p}={S}_{v}-\left({S}_{v}-{P}_{n}\right){\left(\frac{\Delta {t}_{norm}}{\Delta t}\right)}^{x},$$

2 $${S}_{v}={\rho }_{avg}\times g\times z,$$

where *P*_*p*_ is the formation pore pressure (MPa), *S*_*v*_ is the principal vertical stress, *P*_*n*_ is the hydrostatic pore pressure (MPa), Δ*t*_norm_ is the travel time from the normal compaction trend at the given depth (μs), Δ*t* is the observed travel time from the sonic log (μs), *x* is an exponent index, *ρ*_avg_ is the average density of the overburden formation (kg m^−3^), *g* is the acceleration due to gravity (m s^−2^), and *z* is the measured vertical depth (m).

**Figure 2:**
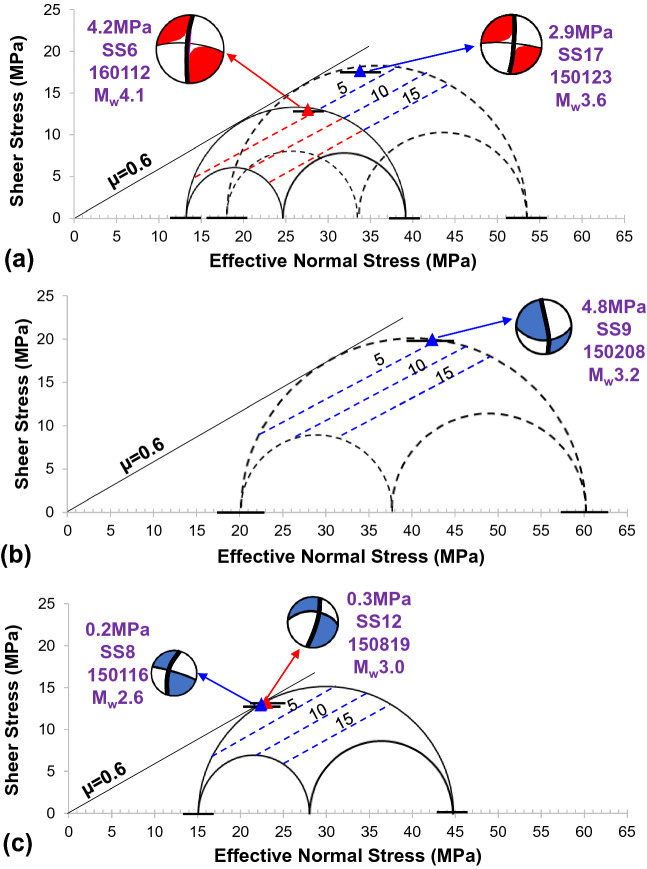
(**a–c**) Mohr circles showing the original stress state of seismogenic faults for five known cases. The contours (units of MPa) within the Mohr circles indicate the increase in pore pressure required to reactivate the associated faults^13,14^. The short black lines in the axis denote the errors of the calculated normal stress.

Shen et al. developed a program for stress calculation in the Kaybob Duvernay region based on a variety of borehole pressure tests (e.g., diagnostic fracture injection test, static gradient survey, and flow/build-up test)^28^. However, only three measurements are available in Shen’s model. In this work, based on Shen’s calculation, we further use the available treatment plot of fracturing wells to supplement the stress calculation in the studied region (see Supplementary Fig. S1). Specifically, the pore pressure is derived from the steady pressure of the last stage in the treatment plot, while the minimum principal stress is estimated from the instantaneous shut-in pressure in the plot. The maximum principal stress is calculated from Zoback’s empirical expression: *S*_*Hmax*_ = 3*S*_*hmin*_ − 2*P*_*p*_ − *P*_*m*_, where *P*_*m*_ is the formation breakdown pressure. Treatment plots of twenty-one wells (blue diamonds in Fig. 1) are employed to estimate the stress data, and the results are obtained (see Supplementary Table S2). The Mohr circles are then plotted to illustrate the stress state of faults related to five mainshocks before HF operations based on the formation and stress calculation results (Fig. 2).

For the SS8 and SS12 cases, an additional increase in pore pressure of 0.2 or 0.3 MPa, respectively, can activate the related seismogenic faults, as both faults are critically stressed. Machine learning-based studies also suggest that the formation overpressure plays an essential role in HF-induced seismicity in the Duvernay shale reservoirs^10^. Therefore, identifying the distribution of formation pore pressure is a significant step in proposing a mitigation strategy for future fracturing operations in the region studied.

#### Distance to the Precambrian basement

**Figure 3:**
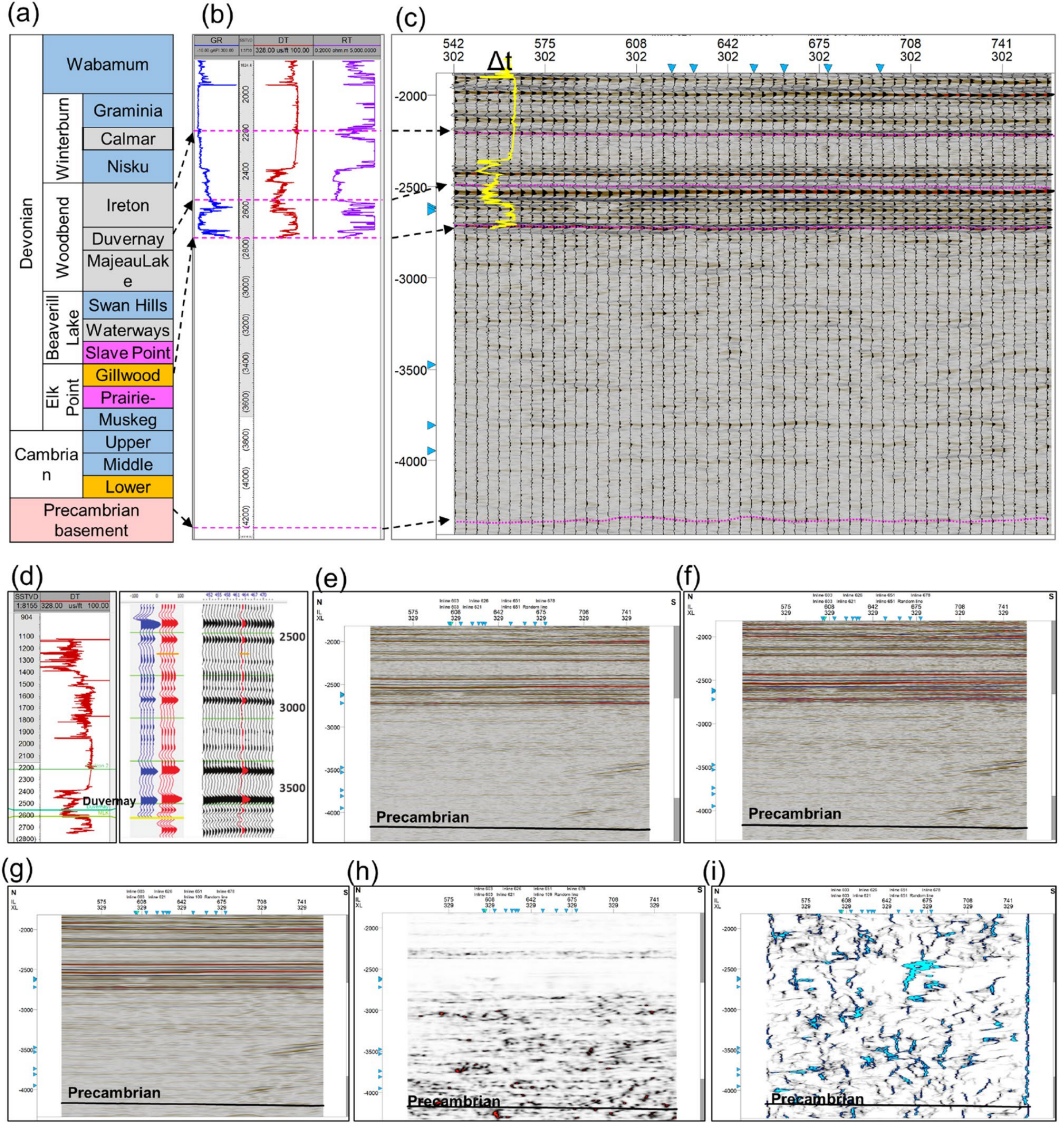
(**a**) Stratigraphy in the region studied. Pink, orange, blue, magenta, and gray represent crystalline, sandstone, limestone, evaporites, and shale rocks^12^. (**b**) Stratigraphy based on data from a coring well (purple well in Fig. 2). (**c**) Cross-section of the 3D seismic survey showing the interpreted Precambrian basement. (**d**) Synthetic seismogram tie of a key well. (**e**) Original seismic profile from 3D seismic survey. (**f**) Trace amplitude grain control. (**g**) Structural smoothing. (**h**) Variance (edge method). (**i**) Ant tracking inversion^30^.

The distance to the Precambrian basement has a vital role in HF-induced seismicity in this region^10^. A shorter distance indicates that a possible flow conduit may exist between the stimulated Duvernay formation and the Precambrian basement, facilitating pressure diffusion along this flow conduit and causing a fault to slip in the Precambrian basement^11^. In this work, the distance to the Precambrian basement was calculated from the vertical distance between the bottom of the Duvernay formation and the top of the Precambrian basement. Specifically, the stratigraphy in this region was investigated based on prior work. As shown in Fig. 3a, the Duvernay formation was deposited in the middle of Devonian sediments, under which the Cambrian and Precambrian basement developed. Then, distinctive logging responses in the Duvernay formation were recognized based on the well-logging features of a straight coring well (Fig. 3b). Because there is no available sonic log in the basement depth, the Precambrian basement is identified from a 3D seismic survey based on the prior works^29^ (Fig. 3c). This approach was applied to well-logging data from other straight wells to obtain the distance to the Precambrian basement at the well site. The distance between the basement and the wells was interpolated with a sequence Gaussian simulation, which was constrained by seismic interpretations. Finally, the distance to the Precambrian basement in the region studied was determined, which provides geological support for the mitigation strategies for HF-induced seismicity.

#### Proximity to pre-existing faults

To determine the distance to a pre-existing fault, it is first important to identify the pre-existing fault. The ant tracking approach based on 3D seismic data has been demonstrated to be applicable for identifying pre-existing faults^30^. Specifically, synthetic seismogram ties of key wells are first established based on P-wave velocity logs and wavelet extraction (Fig. 3d). Then, a trace amplitude grain control step was conducted to scale the instantaneous amplitude with the normalized RMS amplitude over a specified window (Fig. 3e,f). Next, structural smoothing was performed based on a Gaussian weighted filter (Fig. 3g). After that, the variance (edge method) was used to extract an edge volume from the processed seismic volume (Fig. 3h). Finally, ant tracking was conducted to extract faults from a pre-processed seismic volume (Fig. 3i). Pre-existing faults can be identified by the ant tracking method, and so, to mitigate future seismic risks, the proximity of a well to a pre-existing fault can be evaluated.

### Operational control of induced seismicity

#### Safe distance between fracturing wells and potential faults

**Figure 4:**
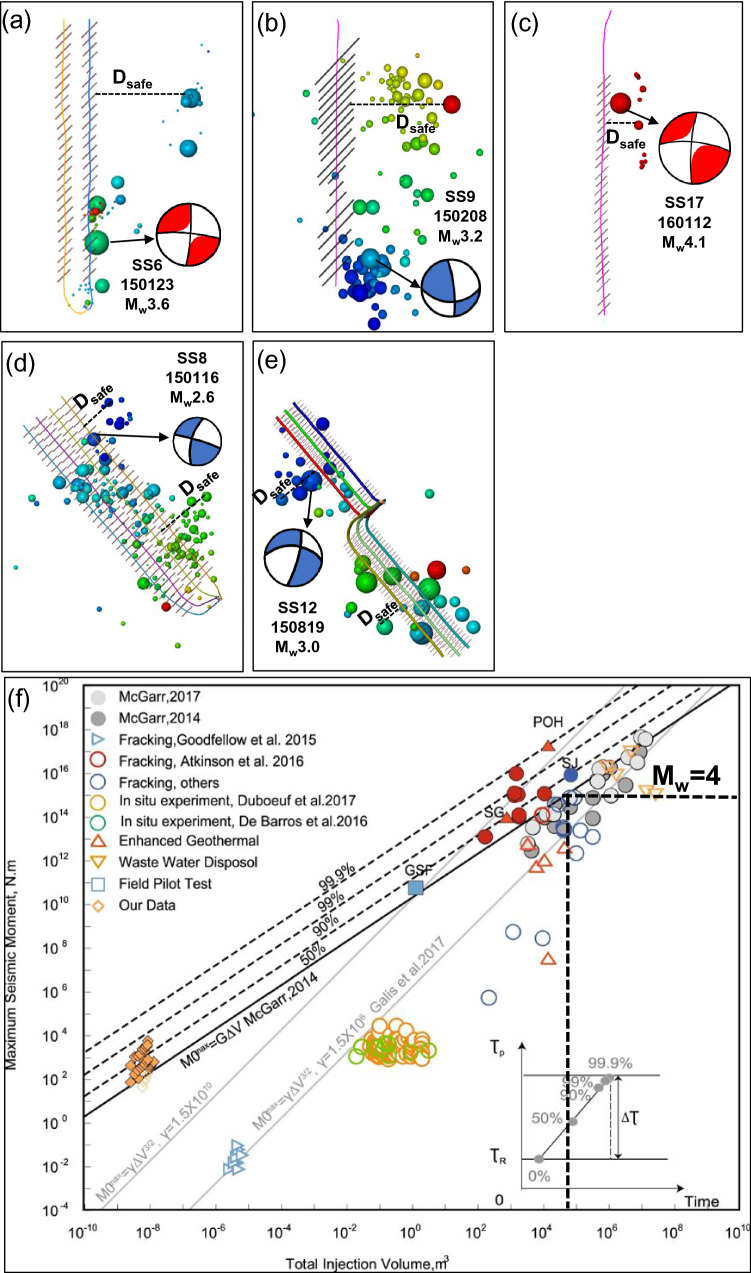
(**a–e**) Maps of fracturing horizontal wells and monitored induced seismicity events for five cases. The fracturing horizontal wells are divided into the N–S-oriented wells (SS6, SS9, and SS17) and NW–SE-oriented wells (SS8 and SS12)^19^. D_safe_ denotes the safe distance between fracturing wells and potential faults. (**f**) Maximum seismic moment vs. total injection volume for fluid-injection-induced earthquakes by Li et al.^34^. The magenta line denotes the possible maximum fluid injection of 89,000 m^3^ with a potential seismicity magnitude of less than 4.0.

Ensuring that there is a moderate distance between a fracturing well and any pre-existing faults can mitigate the risks of seismicity before HF stimulations. In this work, we consider the safe distance from the fracturing site to the pre-existing fault as the furthest induced event (M_L_ > 1.3) with respect to the associated fracturing site of a horizontal well. Here, the local magnitude of 1.3 has been demonstrated to be the magnitude of completeness in this region^3^. Therefore, the furthest induced event with a magnitude larger than 1.3 is regarded as the proxy for the potential fault reactivation. Specifically, we first collected fracturing and seismicity information for five known cases. The distribution of fracturing stages of horizontal wells and the monitored induced seismicity events in the five cases are shown in Fig. 4a–e^19^. Here, we assumed that only one hydraulic fracture was stimulated at each stage and that it propagated along with the NE 45° orientation following the maximum principal stress^30^. Note that fracturing wells in the five cases were divided into N–S-oriented wells (SS6, SS9, and SS17) and NW–SE-oriented wells (SS8 and SS12). Then, the injection volumes of the fracturing fluid for the two types of well in the five cases were plotted versus the distance to the farthest induced events. Finally, a safe well–fault distance can be estimated based on the relationship plots of the maximum seismic moment versus the total injection volume, which was compared with the previous “respect” distance of 895 m between horizontal boreholes and the maximum horizontal stress direction under a strike-slip fault regime^31^. Evaluating the safe HF–fault distance can guide the site selection of horizontal wells in the region studied.

#### Optimizing the fracturing job size

The fracturing job size (e.g., injection volume, pressure, and rate) has been a significant factor contributing to HF-induced seismicity^32–34^. Large volumes of injected fluid can facilitate the diffusion of pressure from the hydraulic fractures upward or downward into the damage zones of seismogenic faults, causing a fault to slip and triggering induced earthquakes.

McGarr developed a formula to calculate the maximum seismic magnitude *M*_0_ (max) from a net injected fluid volume (Δ*V*) for injection-induced earthquakes^32^:

3 $${M}_{0}\left(max\right)=G\Delta V,$$

4 $$G=\frac{E}{2(1+\upsilon )},$$

where *G* is the shear modulus of the medium, *E* is Young’s modulus, and *ν* is Poisson’s ratio.

Equation (3) assumes that: the medium is fully saturated; the medium is in a state of impending failure, and a minimal increase in pressure will cause it to slip; the medium is a Poisson solid; the magnitude vs. frequency distribution has a slope of 1 (*b* = 1)^32–35^.

McGarr’s formula can be used to manage the maximum expected magnitude by limiting the injection volume of the fracturing fluid during HF operations. However, the effects of flowback, together with any interaction between multiple fracturing stages and nearby well pads, can influence the optimization of fluid injection^1^. Li updated a relation plot between a maximum seismic moment and a total injection volume for injection-induced earthquakes based on prior work, as shown in Fig. 4f^34^. Such an updated plot can guide the design of fracturing operations in the studied region.

## Results and discussion

### Geological and operational susceptibility to induced seismicity

#### Geological susceptibility

##### Formation overpressure

**Figure 5:**
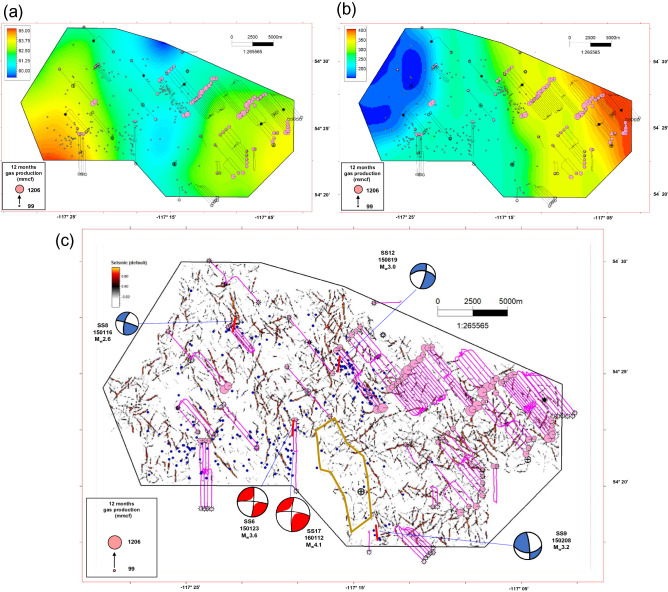
(**a**) Map of formation pore pressure (MPa) in the region studied. The gray and pink circles show the monitored induced earthquakes and 12-month gas production. (**b**) Map of distance (m) to Precambrian basement in the region studied. The gray and pink circles show the monitored induced earthquakes and 12-month gas production. (**c**) Map of pre-existing faults found in the Duvernay formation via the ant tracking approach. The blue circles show the monitored induced earthquakes. The brown polygon marks the optimal fracturing site, where there is a low risk of inducing seismicity.

We used Eqs. (1) and (2) to estimate the formation pore pressure at the well site, which was corroborated by the steady pressure at the end of stage completion of the horizontal wells. Based on the combined analysis, the formation pressure gradient has a range of 17.4–19.2 kPa m^−1^, with a mean value of 18.3 kPa m^−1^ in the region studied (Fig. 5a). These results agree with the mean value of 16.8 kPa m^−1^ found from monitoring tests in previous works^9,10^. Note that the middle and east sections in the region studied have a low degree of formation pressure with a relatively low frequency of induced seismicity, in sharp comparison with the large event in the west section where induced seismicity tends to occur. Therefore, our analysis suggests that the middle and east sections probably have low geological susceptibility to induced earthquakes, based on the expected influence from several of our studied factors.

##### Distance to the Precambrian basement

Based on the logging features and seismic interpretation results at the well site, the distance to the Precambrian basement was determined via a Sequence Gaussian Simulation in the region studied. Based on the aforementioned analysis, the distance to the Precambrian basement is in the range of 154.8–407.0 m, with an average of 273.8 m in the region studied (Fig. 5b). Note that the distance to the Precambrian basement decreases from west to east, corresponding to a seismicity-susceptible region and seismicity-quiescent region, respectively. Therefore, based on the analysis of the distance to the Precambrian basement and induced events, the middle and east sections, which have a distance of more than 250 m, comprise the optimally seismicity-quiescent region for HF operations.

##### Proximity to pre-existing faults

From the results of the ant tracking approach based on the available 3D seismic data, a pre-existing fault network was identified in the region studied. Figure 5c is a map of this fault network with superimposed fracturing horizontal wells and observed induced seismicity events. Note that the strikes of faults inferred via ant tracking matched well with the focal strikes of mainshock events in the five cases (SS6, SS8, SS9, SS12, and SS17), which corroborates the robustness of the fault network inferred via seismic interpretation. Furthermore, the majority of induced events were in the vicinity of the inferred faults, which are concentrated in the west section of the region studied. Although many pre-existing faults were identified in the east section, the induced events in this area rarely occurred during fracturing operations at multistage horizontal wells. This may be because the east section has a relatively low pore pressure (Fig. 5a) and a large distance to the Precambrian basement (Fig. 5b), which both mitigate the risk of seismicity during stimulations^10,11,14^. The brown polygon in Fig. 5c marks the optimal fracturing site, with a low risk of inducing seismicity.

#### Operational susceptibility

##### Safe well-fault distance and wellbore orientation

**Figure 6:**
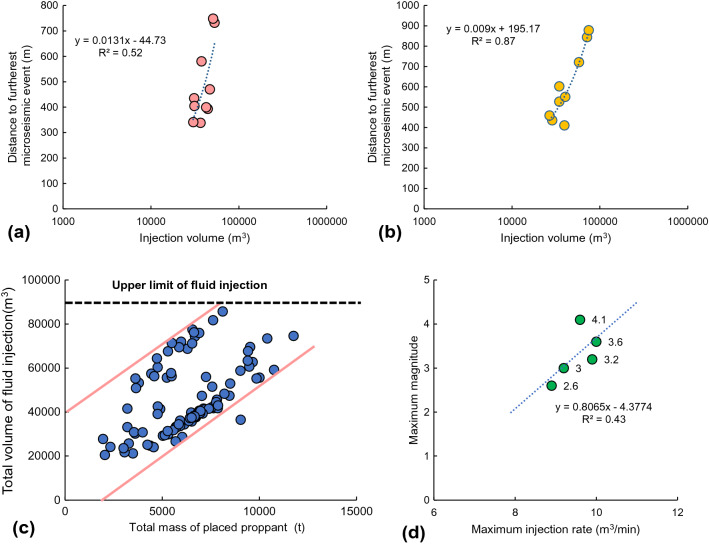
(**a,b**) Distances to the farthest microseismic events vs. fluid injection volumes for N–S-oriented wells and for NW–SE-oriented wells^19^. (**c**) The total volume of fluid injected vs. total mass of placed proppants per well based on the fracturing data for the region studied. (**d**) The maximum magnitude vs. maximum injection rate in five cases.

Figure 6a,b shows the distances to the farthest microseismic events vs. fluid injection volumes for N–S- and NW–SE-oriented wells. The relations between the injection volumes and the distances were different for the two types of horizontal wells. Specifically, the square of the regression coefficient was 0.52 for the N–S-oriented wells, whereas it was 0.87 for the NW–SE-oriented wells. Moreover, the farthest distance (the safe HF–fault distance) was 750 m for a maximum injection volume of 52,820 m^3^ for the N–S-oriented wells, whereas it was 879 m for the NW–SE-oriented wells for a maximum injection volume of 74,485 m^3^. These safe distances are comparable with the result of 895 m in prior works^31^. This moderate distance can guide the selection of fracturing sites before HF operations in the Duvernay shale reservoirs. It is also noted that the safe well-fault distance varies with different fracturing well and associated induced seismicity (i.g., proxies for pre-existing faults) (Fig. 4a–e). In addition, although the magnitude of induced seismicity is empirically proportional to the size of the potential fault^36^, the safe distances are not proportional to the seismicity magnitude (Fig. 4a–e), indicating such safe distances have no linear relationship with the fault size. The in-depth investigation will be conducted in future works to build the poroelastic model of different cases and to simulate the deterministic safe distance under different fault sizes and other site-specific geologic conditions.

##### Optimizing the fracturing job size

The relation between the maximum seismic moment and the total injection volume can be used to determine the maximum fluid injection volume that has a potential seismicity magnitude of less than 4.0 (Fig. 4f). An M_L_ 4.0 event is the red-light threshold for the local traffic light protocol, requiring the causal hydraulic fracturing operation to shut in immediately^7^. From Fig. 4f, it was estimated that the maximum volume of fluid that can be injected is 89,000 m^3^. Additionally, the total volume of fluid injected and total mass of placed proppants per well are plotted with an upper limit for the fluid injection volume of 89,000 m^3^ (Fig. 6c). Therefore, the fluid injection volume of a new fracturing well should be less than 89,000 m^3^ to mitigate the potential seismicity risks. The relationship is also investigated between the maximum seismicity magnitude and maximum injection rate of the associated fracturing well (Fig. 6d). It is found that the injection rate has a linear relationship with the maximum magnitude of induced events. Therefore, the injection rate is recommended to be less than 9.0 m^3^ min^−1^ to avoid an M3.0 event in the studied region.

### Fracturing operations of new wells and related induced seismicity

**Figure 7:**
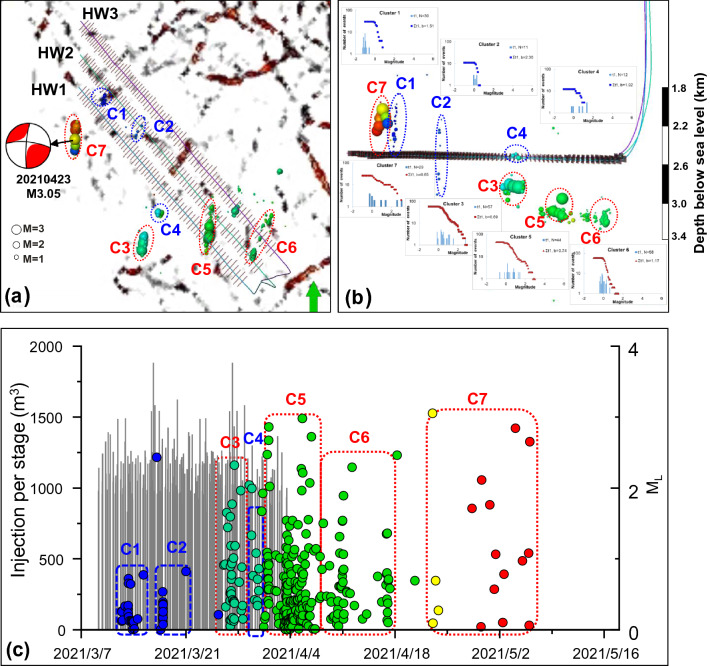
Fracturing operations of three new wells and related induced seismicity. (**a**) Map vies of three fracturing wells superimposed on the pre-existing inferred fault networks. The balls represent the induced earthquakes, colored by time and scaled by magnitude. C1–C7 represent seven earthquake clusters. C1, C2 and C4 were attributed to hydraulic fracture propagation, while the others were linked to reactivation of inferred faults. The beachball shows the focal mechanism solution of the M3.05 event. (**b**) Profile view of HF-induced seismicity and fracturing horizontal wells. Inset maps show the semi-logarithmic magnitude versus frequency distribution for seven clusters. (**c**) Temporal view of treatment data for the three horizontal wells as well as the induced events.

Based on the comprehensive analysis of geological and operational susceptibility to HF-induced seismicity in the region studied, three new horizontal wells were drilled and fractured within the brown polygon in Fig. 5c. From 9 March to 3 April 2021, 225 stage completions {66 for horizontal well 1 (HW1), 75 for horizontal well 2 (HW2), and 84 for horizontal well 3 (HW3)} were performed southward from the toes of three NW–SE-oriented horizontal wells (Fig. 7a). The fluid injection volume and placed proppant for HW1, HW2 and HW3 are 83,411 m^3^ and 12,792 t, 79,921 m^3^ and 13,998 t, 87,056 m^3^ and 15,125 t, respectively.

For the details of the seismicity catalog and focal mechanism inversion, the continuous waveform data were recorded first with a dense shallow buried array during the HF program. Then, a simplified 1D layered velocity model was constructed using sonic-log data from a nearby well, which was then used in both event location and source-mechanism determination processes. Due to the good azimuthal coverage, robust focal-mechanism solutions of induced events were estimated using a similar approach by Zhang et al.^13^, in which 3C displacement amplitudes of direct P-waves are employed. Finally, the induced seismicity catalogs have been obtained (see Supplementary Table S3), and the focal mechanism solution of the mainshock M3.05 has been shown in Fig. 7a. The inversion results are partly validated by the distribution of inferred faults shown in Fig. 7a. Moreover, the focal strike of inferred faults shown in Fig. 7a was in line with the N–S trending of induced seismicity distribution, further corroborating the robustness of focal mechanism solution results.

Based on the results of focal mechanisms, from 3 April to 7 May 2021, 371 induced events with a magnitude range of − 0.3 to 3.05 were recorded around the horizontal wellbores (Fig. 7a,b). Such induced events were distributed in several clusters with a south-north trend (Fig. 7a). More interestingly, the events in the south were induced approximately 200–600 m beneath the stimulated Duvernay Formation, whereas the events in the north were triggered 200–500 m above the Duvernay Formation (Fig. 7b). Such two different distribution patterns of induced events indicate two triggering mechanisms of HF-induced events, the top and basal reactivation of associated inferred faults. Additionally, the latter includes the largest induced event, in this case, *M*_L_ 3.05 event. It was nucleated with a 200-m-offset from the HW1 wellbore on April 23, 20 days after the end of the stage completions. Such a large magnitude event was possibly attributed to the long-term fluid diffusion of fracturing fluids within the fault and hydraulic fracture networks and caused the seismogenic fault to slip. Nevertheless, the issue of trailing seismicity is very complex, which could have been several other mechanisms. Therefore, a delayed pressure migration front is just one possibility. The in-depth investigation will be conducted in the following studies.

The triggering mechanism for Cluster 1–Cluster 7 (C1–C7) has also been investigated. The b value, the slope of the semi-logarithmic magnitude versus frequency distribution, is generally used to distinguish the HF-induced events faults from reactivation-induced events. Based on the large b value (b > 1.5) of C1, C2 and C4 and events distribution surrounding associated hydraulic factures (Fig. 7a,b), such three clusters were possibly attributed to hydraulic fracture propagation. By contrast, C3, C5, C6 and C7, with a relatively low value (b < 1), were located away from related fracturing stages and hence were possibly linked to the reactivation of inferred faults (Fig. 7a,b).

Overall, the fracturing operations of the three horizontal wells were successful. Altogether, 95% of the induced events had a magnitude of less than 2.0. The cumulative gas production was 6394, 7014, and 7213 MMCF within two months for HW1, HW2, and HW3, respectively. Moreover, no induced events were observed after 7 May 2021 (Fig. 7c). This type of comprehensive seismicity risk mitigation is based on integrated data for well completion, well logging, and treatment data and 3D seismic data can be applied to other regions.

### Induced seismic risks assessment and mitigation strategy

#### Definition of an induced seismic risk

Understanding an induced seismic risk is one of the fundamental objectives in earthquake monitoring. Seismic risk is commonly evaluated as a measure for large events that may occur. This is important as it dictates the level of a strong ground motion that may be induced by a seismic event, which is closely related to the potential for damage. A commonly referenced definition of seismic risk is mentioned in prior works as an estimation of the mean probability (over space and time) of the occurrence of a seismic event with a certain magnitude within a given time interval^1,33,35^. The challenges in estimating a seismic risk are clearly highlighted in this definition with regard to the uncertainty involved in “mean probability”, “certain magnitude”, and “within a given time interval”. It is necessary that these three aspects should be considered when assessing seismic risk-related problems.

For mean probability, probabilistic Seismic Hazard Analysis (PSHA) is aimed to quantify the possibility of a ground motion reaching certain arbitrary levels or thresholds at a site when taking all the possible earthquakes (both natural and induced) into consideration^37^. To obtain a robust result of PSHA, understanding the geological background on site is a prerequisite for seismic hazard analysis, which includes formations of rocks, subsurface structures, locations of faults, and a state of stress. A certain magnitude refers to the alert magnitude of induced seismicity regulated by a local regulator. As mentioned in the introduction section, the magnitude of 4.0 is the alert magnitude of the induced earthquake in Alberta. Any operation activities must cease immediately if ML > 4.0 events are nucleated^7^. Within a given time interval refers to a time window with respect to the hydraulic fracturing treatments. Atkinson (2016) adopted a 3-month time window after fracturing completions in the investigation of hydraulic fracturing-induced seismicity in the WCSB^1^.

Here we adopted Shapiro’s occurrence probability model to illustrate the three elements of seismicity risks assessment, “mean probability”, “certain magnitude”, and “within a given time interval”^38^. The expression is shown in Eq. (5), where P(0,M,t) represents the mean probability of the absence of an event with a magnitude larger than a given M in the time interval from 0 (i.e., a start of injection) until t.

5 $$P(0,{\varvec{M}},t)=\text{exp}(-{Q}_{c}\left(t{)10}^{\text{a }-\text{log}\left(\text{Ft} \times \text{S}\right)-\text{b}{\varvec{M}}}\right),$$

where *Q*_*c(t)*_ is the cumulative injected volume at the time at the end of injection; a is a Gutenberg–Richter type statistic value; *Ft* is the tectonic potential, computed by the ratio of the critical maximum pressure parameter, *C*_*max*_, and concentration of pre-existing cracks, N^39^; *S* is the poroelastic uniaxial storage coefficient, constrained to the range of S = 10^–6^ to 0.5 × 10^–7^ m^−1^^40^; *b* is the slope of semi-log plot between seismicity magnitude and frequency.

It is shown that the occurrence probability of events decreases quickly with increasing magnitudes (see Supplementary Fig. S2). Moreover, under the total injection volume of 151,993 m^3^ of fracturing fluid, the observed M_max_ = 3.05 is close to the median prediction of M_max_ = 3.1 while larger than the 95% prediction M_max_ = 1.65. The coincidence between the observed and predicted mean values confirms the accuracy of the probabilistic prediction model (Eq. 3), which can guide the risk evaluation of another fracturing-induced seismicity in this area.

#### Influence of focal mechanisms on the distribution of seismic risks

A focal mechanism is an important feature of a seismic source, which greatly influences the propagation of seismic waves and potential risks. In other words, the ground motions generated by earthquakes are closely related to a focal mechanism^41–45^. Aided by the advanced inversion methods (e.g., full moment tensor inversions), the earthquake source parameters can offer the improved constraints on the spatial locations of a seismicity hypocenter, the geometry of a seismogenic fault^42^, and the state of in-situ stress in a target formation, which is all critical in the assessment of seismic hazard in the WCSB. For example, the inverted magnitude of an event that occurred on 2015/06/13 near the Fox Creek region was 3.93, significantly smaller than the initial report of M_L_ = 4.4. The inverted result indicated that the new position of the event was 10 km away from the initial reported position^41^.

Moreover, due to the available high-resolution monitoring results of seismology stations, the spatial distributions of induced seismicity have also been determined more precisely. For example, for the event clusters that occurred on 2016/01/12 near the Crooked Lake region, Bao and Eaton studied a sequence of these events, including an M3.9 event which occurred several weeks after the related injection^2^. They determined the focal depth of the M3.9 event as 3.9 km, reaching the crystalline basement. However, another study by Eyre et al. utilized a dense, shallow borehole monitoring network for an HF treatment in 2016. They concluded that the majority of the events were located above the target formation, and the magnitude of such events was determined to be M4.1^46^. Therefore, the focal mechanisms from the high-resolution monitoring provided a more robust result.

#### Mitigation and avoidance strategy for HF-induced seismicity

These are two separate strategies for HF-induced seismicity. The first one is avoidance strategy, which is a proactive approach that requires planning and geoscientific assessment prior to fracturing operations. Strategies like increasing a fault-fracture distance are one of the avoidance strategies since operators need to plan to drill around an inventory of known faults. A similar logic applies to earthquake monitoring, stress measurement, geophysical hazard assessments, stimulation fluid design, well/pad orientation, stage spacing, and completion schemas (e.g., single wells and zipper fracks)^6^. The success of the avoidance strategy depends on the quality of available geology, geomechanics and reservoir engineering data, as well as the comprehensive scientific research method.

The second one, mitigation strategy, is generally more reactive. Specifically, it includes measures that are enacted after the induced earthquakes have been encountered. Such strategies include rate/pressure/volume reductions, stage pausing, stage skipping, and as a last resort, well/pad abandonment^6^. However, some hydraulic fracturing-induced seismicity owns a feature of hysteresis. Based on some previous studies, some large magnitude earthquakes were triggered several days, even months, after all stage completions of fracturing wells^14^, making the mitigation strategy not effective and timely.

However, we also noticed that specific to Alberta/WCSB, the fracking cases that caused these earthquakes currently being studied were found to be in excess of those predicted by the McGarr or Li relationship, suggesting that this relationship may not be useful in WCSB. In other words, M4 + events have already happened in Duvernay using a smaller volume than the authors recommended as the cap to prevent the red-light events (Fig. 6c). To date, we have not figured out another relatively precise plot or equations to guide the fracturing design in terms of fracturing fluid injection, which will be further investigated in our future studies.

## Concluding remarks

In this paper, a comprehensive investigation of risk mitigation for HF-induced seismicity was conducted based on field cases near Crooked Lake. Data from well completion, well logging, and core experiments of associated wells and regional 3D seismic data were collected as integrated datasets. Based on the spatiotemporal features of the induced seismicity and real-time treatment data from fracturing horizontal wells, an in-depth investigation of geological susceptibility and operational susceptibility was performed. Finally, new wells were drilled and fractured with optimal fluid injection within the safe region, which has a low risk of seismicity. Our conclusions are as follows:

1. The gradient of the formation pressure has a range of 17.4–19.2 kPa m^−1^, with a mean value of 18.3 kPa m^−1^ in the region studied. The middle and east sections comprise an optimally seismicity-quiescent region for fracturing operations.
2. The distance to the Precambrian basement is in the range of 154.8–407.0 m, with an average of 273.8 m in the region studied. There is a declining trend from west to east. The middle and east sections, which have a distance of more than 250 m, comprise the optimally seismicity-quiescent region for HF operations.
3. The pre-existing fault network was identified in the region studied from the results of the ant tracking approach based on the available 3D seismic data. The south-central region has a low fault density, indicating that this region is a low likelihood of causing induced earthquakes if drilling/stimulation was to perform in this region.
4. The safe HF–fault distance was 750 m for a maximum injection volume of 52,820 m^3^ for the N–S-oriented wells, whereas it was 879 m for a maximum injection volume of 74,485 m^3^ for the NW–SE-oriented wells.
5. According to the relation between the maximum seismic moment and the total injection volume, the fluid injection volume for a new fracturing well should be less than 89,000 m^3^ to mitigate the risk of potential seismicity.
6. The fracturing operations of three new horizontal wells were successful. Altogether, 95% of the induced events had a magnitude of less than 2.0, and the production performance was high, demonstrating the applicability of this comprehensive approach for seismicity risk mitigation.

## Supplementary Information

Supplementary Information.

## Data Availability

The induced seismicity catalog is obtained from the Composite Alberta Seismicity Catalogue (www.inducedseismicity.ca/catalogues, last accessed on 2021/09/01). The well logging, completion, experiments, and production data are sourced from the geoLOGIC database.
