# Supplementary material for: Mitigating risks from hydraulic fracturing-induced seismicity in unconventional reservoirs: case study
Source: Sci Rep. 2022 Jul 22;12:12537. doi: 10.1038/s41598-022-16693-3 (PMC9307861; doi:10.1038/s41598-022-16693-3)
Supplement: Supplementary file 1 — Supplementary Information. [file 41598_2022_16693_MOESM1_ESM.docx]

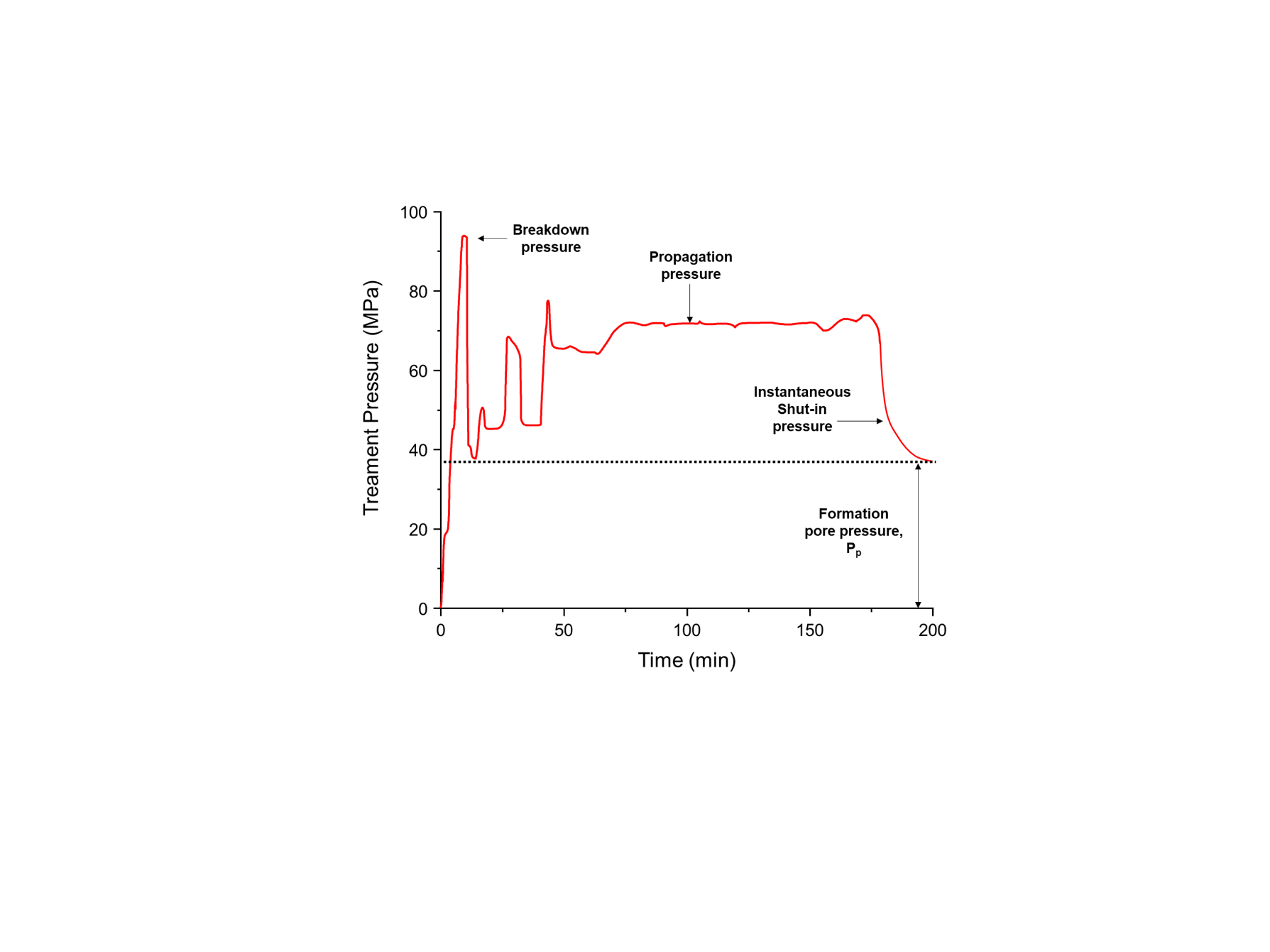


**Supplementary Figure S1.** The treatment plot of one-stage completion showing the estimation of pressure and stress. The pore pressure is derived from the steady pressure of the last stage, while the minimum principal stress is estimated from the instantaneous shut-in pressure.


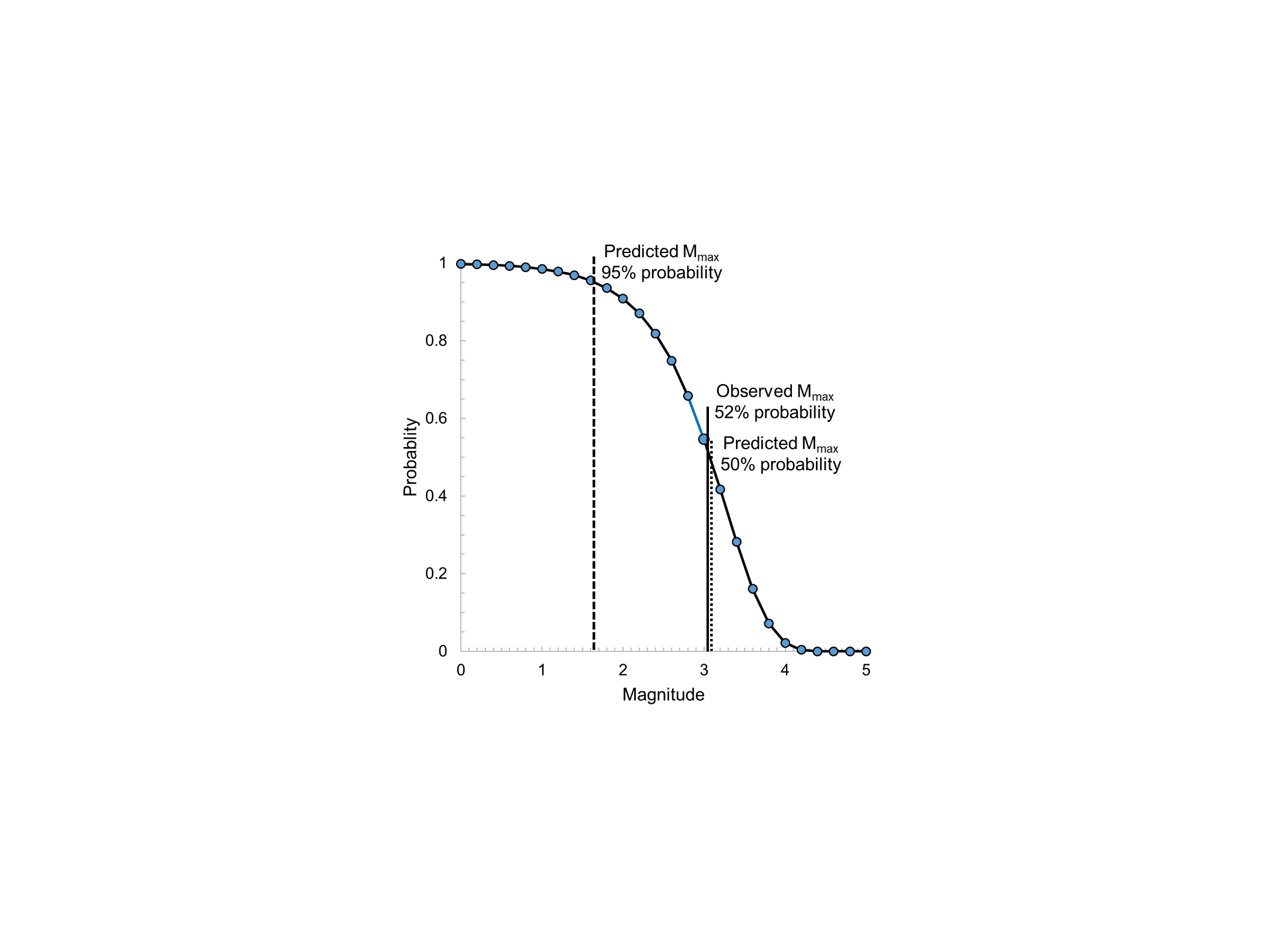


**Supplementary Figure S2.** Comparison of Shapiro et al. (2010) predicted M_max_ and observed M_max_ for the studied case. The predicted M_max_ with 50% and 95% probability are M1.65 and M3.1, respectively. The observed M_max_ M3.05 corresponds to the 52% probability.

**Supplementary Table S1.** Experimental results of core samples from two coring wells (green triangles in Figure 1b)

| Wells | Sample ID | Core Depth (m) | Bulk Density (g/cm^3^) | Effective Porosity (%) | Pressure-Decay  Permeability  (mD) | Effective  Gas Saturation (% ) | TOC (% ) | Shale content (% ) |
| --- | --- | --- | --- | --- | --- | --- | --- | --- |
| CW1 | 1 | 3299.66 | 2.437 | 7.41 | 1.7500E-04 | 73.8 | 2.75 | 14 |
| CW1 | 2 | 3310.36 | 2.503 | 3.24 | 1.3578E-04 | 53.4 | 3.5 | 50 |
| CW1 | 3 | 3313.94 | 2.454 | 3.28 | 1.5482E-04 | 53.0 | 4.55 | 21 |
| CW1 | 4 | 3318.06 | 2.449 | 4.17 | 2.3409E-04 | 61.9 | 3.95 | 27 |
| CW2 | 1 | 3299.66 | 2.437 | 7.41 | 1.7500E-04 | 73.8 | 2.14 | 38.3 |
| CW2 | 2 | 3423.31 | 2.618 | 3.14 | 1.0299E-04 | 78.22 | 2.19 | 40 |
| CW2 | 3 | 3424.42 | 2.539 | 3.30 | 1.3487E-04 | 59.98 | 3.28 | 42.1 |
| CW2 | 4 | 3425.07 | 2.554 | 2.86 | 1.2550E-04 | 56.46 | 3.02 | 30.9 |
| CW2 | 5 | 3425.63 | 2.524 | 3.04 | 1.2476E-04 | 51.73 | 3.45 | 35.4 |
| CW2 | 6 | 3427.09 | 2.543 | 2.79 | 1.0909E-04 | 50.51 | 2.69 | 38.2 |
| CW2 | 7 | 3428.09 | 2.603 | 2.92 | 1.0451E-04 | 64.84 | 2.15 | 27.1 |
| CW2 | 8 | 3428.51 | 2.514 | 3.26 | 1.1212E-04 | 48.54 | 3.58 | 37.6 |
| CW2 | 9 | 3429.59 | 2.526 | 3.73 | 1.1315E-04 | 53.69 | 2.65 | 26.6 |
| CW2 | 10 | 3430.80 | 2.512 | 3.61 | 1.6573E-04 | 60.53 | 3.18 | 22.9 |
| CW2 | 11 | 3432.06 | 2.470 | 8.06 | 7.1310E-05 | 29.42 | 2.32 | 13 |
| CW2 | 12 | 3432.48 | 2.480 | 5.19 | 6.4112E-05 | 45.38 | 2.95 | 21 |
| CW2 | 13 | 3433.06 | 2.504 | 4.41 | 1.4565E-04 | 48.27 | 3.01 | 21 |
| CW2 | 14 | 3433.27 | 2.492 | 4.41 | 1.2613E-04 | 50.93 | 2.71 | 27 |
| CW2 | 15 | 3434.06 | 2.542 | 3.93 | 1.1618E-04 | 66.34 | 3.14 | 42.5 |
| CW2 | 16 | 3434.83 | 2.535 | 3.87 | 1.1283E-04 | 42.75 | 2.78 | 51.1 |
| CW2 | 17 | 3435.44 | 2.541 | 3.46 | 1.1585E-04 | 48.28 | 2.46 | 42.4 |
| CW2 | 18 | 3436.94 | 2.530 | 4.38 | 1.0140E-04 | 59.50 | 2.45 | 36 |
| CW2 | 19 | 3437.96 | 2.535 | 3.87 | 1.1545E-04 | 62.72 | 3.17 | 35.6 |
| CW2 | 20 | 3438.77 | 2.521 | 2.95 | 6.6110E-05 | 46.95 | 3.28 | 38 |
| CW2 | 21 | 3439.17 | 2.519 | 3.28 | 5.6716E-05 | 51.49 | 3.3 | 38.4 |
| CW2 | 22 | 3441.09 | 2.515 | 3.39 | 3.3257E-05 | 60.26 | 3.23 | 38.1 |
| CW2 | 23 | 3441.66 | 2.505 | 3.31 | 1.2463E-04 | 54.21 | 3.88 | 37 |
| CW2 | 24 | 3442.77 | 2.508 | 2.80 | 1.2733E-04 | 57.94 | 3.9 | 42.6 |
| CW2 | 25 | 3443.14 | 2.491 | 3.43 | 1.5182E-04 | 46.82 | 4.16 | 27.6 |
| CW2 | 26 | 3445.10 | 2.484 | 3.15 | 1.2325E-04 | 45.74 | 4.18 | 25 |
| CW2 | 27 | 3445.69 | 2.467 | 3.31 | 1.5574E-04 | 52.70 | 4.32 | 21.2 |
| CW2 | 28 | 3450.03 | 2.471 | 3.26 | 1.7045E-04 | 62.22 | 4.25 | 21 |
| CW2 | 29 | 3451.02 | 2.485 | 3.95 | 1.6069E-04 | 59.50 | 3.21 | 22.8 |
| CW2 | 30 | 3452.34 | 2.496 | 3.80 | 1.5544E-04 | 61.44 | 3.6 | 35.6 |
| CW2 | 31 | 3453.33 | 2.478 | 4.28 | 1.6460E-04 | 66.30 | 3.21 | 27.2 |
| CW2 | 32 | 3453.56 | 2.467 | 5.15 | 1.6760E-04 | 69.00 | 3.28 | 30.3 |
| CW2 | 33 | 3454.17 | 2.476 | 4.80 | 1.7680E-04 | 69.72 | 3.42 | 31.9 |
| CW2 | 34 | 3454.67 | 2.441 | 5.13 | 1.6431E-04 | 69.37 | 4.74 | 30.6 |
| CW2 | 35 | 3455.07 | 2.484 | 4.13 | 1.4625E-04 | 60.57 | 3.53 | 32.4 |
| CW2 | 36 | 3455.26 | 2.436 | 4.28 | 1.6061E-04 | 65.70 | 4.72 | 33.6 |
| CW2 | 37 | 3455.85 | 2.556 | 2.96 | 1.4345E-04 | 51.05 | 2.98 | 35.3 |
| CW2 | 38 | 3455.93 | 2.456 | 3.62 | 1.4075E-04 | 57.85 | 3.93 | 42 |
| CW2 | 39 | 3456.55 | 2.482 | 4.06 | 1.5051E-04 | 58.86 | 3.98 | 17.5 |
| CW2 | 40 | 3458.01 | 2.489 | 3.91 | 1.7125E-04 | 59.62 | 3.8 | 27.9 |
| CW2 | 41 | 3459.52 | 2.501 | 4.18 | 1.7294E-04 | 64.60 | 2.59 | 42.2 |
| CW2 | 42 | 3461.14 | 2.678 | 0.74 | 5.6903E-05 | 95.99 | 0.53 | 33.9 |
| CW2 | 43 | 3462.03 | 2.678 | 1.04 | 6.2859E-05 | 79.01 | 0.5 | 21.1 |

**Supplementary Table S2.** The in-situ stress and pore pressure estimation for fracturing wells (blue diamonds in Figure 1b)

| Name | S_hmin_ | P_p_ | S_Hmax_ |
| --- | --- | --- | --- |
|  | MPa | MPa | MPa |
| W1 | 79.6 | 61.8 | 105.0 |
| W2 | 80.4 | 61.9 | 107.3 |
| W3 | 78.8 | 63.3 | 99.7 |
| W4 | 81.2 | 63.9 | 105.9 |
| W5 | 76.8 | 64.4 | 91.5 |
| W6 | 80.2 | 63.0 | 104.8 |
| W7 | 78.7 | 61.4 | 103.3 |
| W8 | 79.5 | 61.2 | 106.1 |
| W9 | 81.2 | 61.0 | 111.5 |
| W10 | 78.0 | 61.2 | 101.5 |
| W11 | 76.4 | 61.2 | 96.7 |
| W12 | 80.4 | 61.5 | 108.2 |
| W13 | 79.6 | 61.8 | 105.1 |
| W14 | 78.8 | 61.8 | 102.6 |
| W15 | 76.9 | 63.0 | 94.5 |
| W16 | 75.2 | 62.4 | 90.9 |
| W17 | 70.1 | 63.0 | 74.3 |
| W18 | 69.0 | 62.9 | 71.2 |
| W19 | 69.9 | 62.3 | 75.0 |
| W20 | 69.9 | 61.7 | 76.3 |
| W21 | 79.3 | 62.7 | 102.5 |

**Supplementary Table S3.** The catalog of induced seismicity (M_L_>2.0) in the studied case

| Event ID | Time | Latitude | Longitude | Depth (km) | Magnitude | Magnitude type |
| --- | --- | --- | --- | --- | --- | --- |
| 1 | 2021/5/5 19:39 | 54.42 | -117.28 | 3.22 | 2.65 | ML |
| 2 | 2021/5/3 20:58 | 54.41 | -117.28 | 3.19 | 2.84 | ML |
| 3 | 2021/4/29 8:36 | 54.41 | -117.28 | 3.14 | 2.11 | ML |
| 4 | 2021/4/22 19:15 | 54.41 | -117.28 | 3.09 | 3.05 | ML |
| 5 | 2021/4/18 0:40 | 54.41 | -117.28 | 3.14 | 2.46 | ML |
| 6 | 2021/4/12 0:15 | 54.41 | -117.31 | 2.92 | 2.29 | ML |
| 7 | 2021/4/6 13:24 | 54.40 | -117.25 | 3.73 | 2.72 | ML |
| 8 | 2021/4/6 8:18 | 54.40 | -117.25 | 3.74 | 2.07 | ML |
| 9 | 2021/4/5 9:45 | 54.40 | -117.25 | 3.74 | 2.21 | ML |
| 10 | 2021/4/5 8:34 | 54.40 | -117.25 | 3.64 | 2.98 | ML |
| 11 | 2021/4/5 5:11 | 54.40 | -117.25 | 3.75 | 2.27 | ML |
| 12 | 2021/3/31 23:54 | 54.40 | -117.24 | 3.79 | 2.02 | ML |
| 13 | 2021/3/31 21:13 | 54.40 | -117.26 | 3.53 | 2.96 | ML |
| 14 | 2021/3/31 20:51 | 54.40 | -117.26 | 3.53 | 2.67 | ML |
| 15 | 2021/3/31 15:44 | 54.40 | -117.26 | 3.53 | 2.47 | ML |
| 16 | 2021/3/29 6:28 | 54.40 | -117.26 | 3.53 | 2.04 | ML |
| 17 | 2021/3/27 6:22 | 54.40 | -117.26 | 3.52 | 2.32 | ML |
| 18 | 2021/3/16 20:13 | 54.41 | -117.28 | 3.18 | 2.43 | ML |
